# Of woods and webs: possible alternatives to the tree of life for studying genomic fluidity in *E. coli*

**DOI:** 10.1186/1745-6150-6-39

**Published:** 2011-07-20

**Authors:** Julie Beauregard-Racine, Cédric Bicep, Klaus Schliep, Philippe Lopez, François-Joseph Lapointe, Eric Bapteste

**Affiliations:** 1 Département de sciences biologiques, Université de Montréal, Montréal (Québec), Canada; 2 UMR CNRS 7138 Systématique, Adaptation, Evolution, Université Pierre et Marie Curie, 75005 Paris, France

**Keywords:** *E. coli*, trees, networks, quartets, lateral gene transfer, methodological pluralism

## Abstract

**Background:**

We introduce several forest-based and network-based methods for exploring microbial evolution, and apply them to the study of thousands of genes from 30 strains of *E. coli*. This case study illustrates how additional analyses could offer fast heuristic alternatives to standard tree of life (TOL) approaches.

**Results:**

We use gene networks to identify genes with atypical modes of evolution, and genome networks to characterize the evolution of genetic partnerships between *E. coli *and mobile genetic elements. We develop a novel polychromatic quartet method to capture patterns of recombination within *E. coli*, to update the clanistic toolkit, and to search for the impact of lateral gene transfer and of pathogenicity on gene evolution in two large forests of trees bearing *E. coli*. We unravel high rates of lateral gene transfer involving *E. coli *(about 40% of the trees under study), and show that both core genes and shell genes of *E. coli *are affected by non-tree-like evolutionary processes. We show that pathogenic lifestyle impacted the structure of 30% of the gene trees, and that pathogenic strains are more likely to transfer genes with one another than with non-pathogenic strains. In addition, we propose five groups of genes as candidate mobile modules of pathogenicity. We also present strong evidence for recent lateral gene transfer between *E. coli *and mobile genetic elements.

**Conclusions:**

Depending on which evolutionary questions biologists want to address (i.e. the identification of modules, genetic partnerships, recombination, lateral gene transfer, or genes with atypical evolutionary modes, etc.), forest-based and network-based methods are preferable to the reconstruction of a single tree, because they provide insights and produce hypotheses about the dynamics of genome evolution, rather than the relative branching order of species and lineages. Such a methodological pluralism - the use of woods and webs - is to be encouraged to analyse the evolutionary processes at play in microbial evolution.

This manuscript was reviewed by: Ford Doolittle, Tal Pupko, Richard Burian, James McInerney, Didier Raoult, and Yan Boucher

## Background

For a long time, the reconstruction of the tree of life (TOL) was an important goal of evolutionary science [1]. This inclusive hierarchical classification, through its genealogical structure, was expected to reflect the relative branching order of all biological lineages, as they diverged from a last common ancestor. This unique, universal, natural, and genealogical pattern was therefore invested with important practical and heuristic powers [2,3]. The TOL became central in attempts to make sense of the huge diversity of forms and adaptations produced during evolution. It was in particular considered to be the most important of all phylogenetic objects, since it provided the best backbone to map the origins of lineages, biological features and their subsequent modifications.

In order to successfully reconstruct the TOL, homologous characters, comparable among all life forms, were needed. Genes and proteins appeared to be ideal materials for retracing evolution at both large and small evolutionary scales, since the vast majority of evolving entities harbour these materials, and they can therefore be compared widely. However, due to the limited size of individual genes and the importance of horizontal transfer of DNA, the strength of the phylogenetic signal in single molecules was often too low to resolve the entire TOL. Multiple phylogenetic markers, in fact multiple genes, were therefore used to propose a well resolved TOL, either by the concatenation of markers, by averaging their phylogenetic signal, or by a corroboration of their individual phylogenetic signals in congruence analyses that sought a hierarchical pattern shared by most of these genes [2,4,5].

Yet, doubts were legitimately raised about the relevance (meaning and feasibility) of these various multi-gene approaches. First, if there are several major evolutionary transitions (e.g., from a pre-DNA to a DNA-based genetic system, etc.), homology in the genes might not be a sufficient guideline to describe early evolution. Second, doubts were raised because these approaches were clearly designed to subsume the history of the multiple markers under one overarching (or an average) phylogenetic history [1,6,7]. The recognition that individual genes - even from a given genome - often had uncoupled evolutionary histories, at the very least for prokaryotes and for mobile elements, prompted questioning about whether a single (dominant/average or most corroborated) tree-like phylogenetic pattern was the most suitable representation of evolution [8-21]. Rather than producing a satisfactory TOL, phylogenomic analyses based on multiple genes generated a massive phylogenetic forest of gene trees [4,22,23]. Many of these gene trees displayed different topologies, not only due to tree reconstruction artefacts, but also due to lateral gene transfer (LGT), gene losses and gene duplications [5,24-30].

Simply put, it became clear that independent processes had impacted the evolutionary history of genes and genomes, and therefore of the lineages under study in prokaryotes and mobile elements, and that evolution had followed a more complex pattern than anticipated by Darwin and subsequent evolutionists. Indeed, prokaryotes and mobile elements represent and have always represented the vast majority of life [31-33]. This realization had some impact on phylogenetics, which had historically considered evolution through the lens of systematics rather than ecology. Core genes, often assumed to be vertically inherited, were typically expected to produce a fundamental vertical framework, against which the evolution of traits and lineages was to be interpreted. Such core genes appeared suited to think about "groups within groups", which is a logic consistent with systematics. However, the distribution of shell genes was clearly explained by additional evolutionary processes, involving in particular gene transfers between partners with overlapping lifestyles or environments. Most of gene evolution (that of shell genes) appeared therefore better interpreted in light of an ecological vision. Some evolutionists were reluctant to consider a different model than the TOL to study the multiple processes and the distinct outcomes of evolution in more details, but many acknowledged by changing their practices that phylogenetic research required some adjustment [22,23,28,34-37].

In particular, some researchers proposed reconstructing phylogenetic networks, rhizomes or syntheses of life instead of a strict tree, making it possible to distinguish the vertical backbone (tracking the lineage of dividing cells) from horizontal transfers, which were represented by additional lateral branches. These new methods produced a more complex representation that could account for both genealogy and horizontal transfer [13,34,36-39].

The decision to pursue this novel objective testifies that the ultimate phylogenetic object of evolutionary analysis, traditionally a common bifurcating tree, can change. Yet, it is worth debating whether the particular solution of a "banyan tree" based on multiple markers is the only valuable result of evolutionary analyses [12,16,21,40]. This kind of phylogenetic networks emphasized the fact that evolutionary patterns are caused by independent processes impacting the evolutionary histories of genes, i.e. that there is often more than one process at play. From a pluralistic perspective, methods specifically designed to reveal the multiple processes behind the pattern are necessary, as they challenge attempts to explain all patterns by a single process (e.g. all evolution by a tree-like process of descent). A tree alone is not going to help establish much of this evolutionary complexity.

It is striking that today's primary material for evolutionary studies is itself a new phylogenetic object: a large forest of life (FOL) [4,22]. This observation opens the doors to pluralistic and pragmatic developments in the research program of phylogenetics (or, as some might say, to post-phylogenetic evolutionary research programs). Depending on what evolutionary questions are to be addressed, many possible approaches can be used to harvest the FOL [22,23,41,42], without giving an absolute priority to the reconstruction of the TOL (perceived as a statistical trend or as the real genealogy of evolving entities). Moreover, other representations than the FOL, for instance those based on networks [18-21,41,43,44], can be used to address distinct evolutionary questions, at different biological scales.

In this work, we use 141,493 genes of 30 strains of *E. coli*, 300,841 genes from 119 prokaryotic genomes (54 archaea, 65 bacteria) and 228,131 genes from mobile elements to illustrate that interesting questions about evolution can be tackled, and new knowledge can be produced, with new methods/tools that go beyond the TOL. More precisely, we illustrate the genetic mosaicism of *E. coli *[25,45] and some of its causes with two methods of shared sequence network analysis (the genome network [21] and the gene network [43]) and with two methods for harvesting the FOL (clanistic analysis [41,42], and a novel approach based on polychromatic quartets: PQ). These methods of evolutionary analyses unravel a bit more how *E. coli *adapted to their environments.

## Results and Discussion

### A few lessons from networks

#### Using genome networks to detect recent LGT in the *E. coli *pangenome

Genome networks are shared sequence networks that display the overlap in genetic content between genomes [13,18,21]. Nodes of genome networks correspond to genomes, connected by weighted edges that are inversely proportional to the number of homologous families these genomes share. Such networks are excellent tools to unravel patterns of gene sharing caused by conjugation and transduction events that result in shared DNA material between chromosomes and the genomes of plasmids, and between chromosomes and the genomes of phages, respectively. In our genome network, we focused on the genetic interactions between *E. coli *and the mobile elements, and their evolution over time. Indeed, such connections suggest which gene families - and how many - may have been introduced in the chromosomes of *E. coli *by mobile elements, or may have moved from these chromosomes to the genomes of mobile elements.

**Figure 1:**
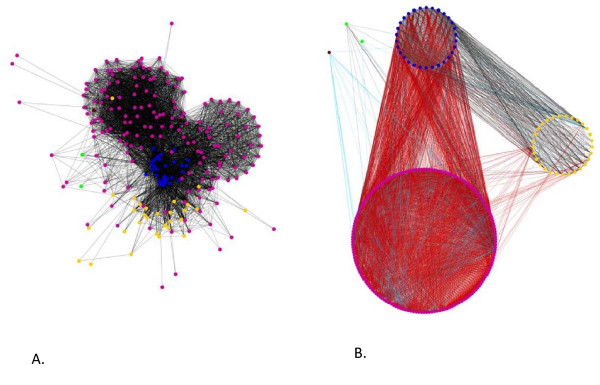
**Genome network of *E. coli *at 100% identity**. (A) Each node corresponds to a genome (blue for *E. coli*, purple for plasmid, orange for viruses, brown for *E. histolytica*, green for *A. laidlawii *and *S. putrefaciens*). Edges connect pairs of genomes sharing at least one gene with 100% identical sequence. The display is a spring-embedded layout. (B) Same dataset and same colour code for the nodes. The display was a group attributes layout, with three groups: viruses, plasmids and *E. coli*. Edges are coloured based on the dominant function of the shared genes: red for the replication and repair category, cyan for all the other COG categories and black for genes without known functions. Cytoscape [66] was used for both displays.

In order to find such candidate gene families "recently moved", we divided the genome network into slices and focused on shared gene families displaying 100% sequence identity between *E. coli *and the mobile elements (Figure 1A). We observed that 170 plasmids and 29 viruses harboured sequences from 416 gene families 100% identical with *E. coli*. Such a similarity is unlikely to be the result of a very strong purifying selection that has been constantly exerted on these sequences. Rather, it may correspond to recent exchanges between *E. coli *and the mobile elements. Therefore, in our gene network, *E. coli *appears at the center of a cloud of mobile DNA, as these cellular genomes are surrounded by mobile genetic elements with which they interact. The interaction presented in this type of evolutionary representation demonstrates beyond doubt that *E. coli *and mobile genetic elements mutually affect each other's recent evolution. They are partners, consistent with previous findings [46] that highlight the role of huge viral populations in providing adaptive genes to their cellular hosts in the digestive tract.

Interestingly, 42% of these 4361 sequences belonged to the L functional categories (Replication and repair) (Table 1). These particular sequences were thus likely to be involved in the lateral transfer itself, and as such may be considered as strong evidence for these recent LGTs. Through this analysis, not only the vectors can be identified but also the genes that played a role in the insertion of exogenous DNA material (Figure 1B). Interestingly, when particular plasmids and viruses shared such sequences for replication and repair with *E. coli*, they also often shared additional sequences, from other functional categories. Replication and repair sequences may have helped to move these other sequences around. Moreover, while both viruses and plasmids transferred such replication and repair sequences, most of the genes shared between viruses and *E. coli *were - remarkably - of unknown function (Figure 1B). Another 42% of the "recently" transferred sequences had unknown functions. The results were less dramatic but similar when expressed in number of families shared between *E. coli *and mobile elements: 61% had unknown functions, and 16% belonged to the replication and repair category.

**Table 1:** Number of sequences and gene families in the genome networks, classified by functional categories

	COG	100%-seq	90-99%-seq	100%-fam	90-99%-fam
**Information, storage, processing**					

RNA processing and modification	A	0	0	0	0

Chromatin structure and dynamics	B	0	0	0	0

Translation, ribosomal structure and biogenesis	J	28	120	2	4

Transcription	K	131	255	18	27

Replication, recombination and repair	**L**	**1838**	**3670**	**68**	**94**

**Cellular Processes and Signalling**					

Cell cycle control, cell division, chromosome partitioning	D	12	15	1	1

Signal transduction mechanisms	T	82	294	11	24

Defense mechanisms	V	22	148	3	9

Nuclear structure	Y	0	0	0	0

Cell wall/membrane/envelope biogenesis	M	56	295	5	13

Cell motility	N	3	69	1	5

Extracellular structures	W	0	0	0	0

Cytoskeleton	Z	0	0	0	0

Posttranslational modification, protein turnover, chaperones	O	0	50	0	4

Intracellular trafficking, secretion, and vesicular transport	U	5	94	2	13

**Metabolism**					

Energy production and conversion	C	0	56	0	5

Amino acid transport and metabolism	E	6	40	2	6

Nucleotide transport and metabolism	F	0	28	0	1

Carbohydrate transport and metabolism	G	23	159	6	8

Coenzyme transport and metabolism	H	49	3	2	1

Lipid transport and metabolism	I	0	13	0	1

Inorganic ion transport and metabolism	P	67	371	4	21

Secondary metabolites biosynthesis, transport and catabolism	Q	16	56	2	4

**Poorly characterized**					

General function prediction only	R	120	806	19	52

Function unknown	S	138	885	20	45

Unknown by RPS-Blast	**X**	**1832**	**4589**	**260**	**356**

The functional categories are the COG categories, assigned by RPS-BLAST. The two first left columns of data indicate the number of sequences, and the remaining two columns indicate the number of gene families, in the 100% identity genome network (first), and in the 90-99% identity genome network (second).

This important co-evolutionary interaction between *E. coli *and mobile elements concerns not only "recent" periods of time. The analyses of other slices of the genome network (when the identity threshold between homologs in *E. coli *and mobile genetic elements was relaxed, i.e. when families shared between *E. coli *and mobile elements with 90-99% identity were investigated), we obtained a similar picture. In that slightly more "ancient" genome network, *E. coli *shared genetic material with 249 plasmids and 40 viruses from 673 gene families. Sequences involved in replication and repair were still very detectable (30.5% of the sequences and 13.5% of the gene families), and the proportion of sequences without known function, although still dominant, slightly decreased in these 11,805 sequences (38.2% of the sequences, 51.3% of the gene families) (Table 1). Overall these results show the important cumulative effect that LGT can have on microbial genomes.

In addition, these genome networks highlighted that *E. coli *shared some sequences that were 90-100% identical with two pathogenic bacterial genomes (one IS-10 transposase with *Acholeplasma laidlawii*, and nine genes with *Shewanella putrefaciens*: namely a heavy metal translocating P-type ATPase, a 30S ribosomal protein S12, a hypothetical protein Sputcn32_0263, a copper/silver efflux system membrane fusion protein CusB, a transposase, IS4 family protein, the IS630 ORF, a peptidase M23B, a DNA-binding transcriptional activator CusR, a sensor kinase CusS, a CzcA family heavy metal efflux protein, an insertion element protein, and a periplasmic copper-binding protein), and with one intestinal eukaryote (an aminoglycoside 3'-phosphotransferase with *Entamoeba histolytica*). Whether these cases are real lateral transfer between these organisms, or contamination, may be worth investigating in future studies.

#### *E. coli *gene networks: a brief look at the diverse evolutionary modes affecting gene families

We also used gene networks to rapidly investigate the evolution of genetic diversity of homologous families within pathogens and non-pathogens, with a focus on *E. coli*. Unlike the genome network, a gene network [43] has gene sequences at its nodes, instead of genomes. Sequences are connected by weighted edges when they share a relationship of homology/identity, as assessed by a BLAST search. Each gene family is therefore easily characterized because it falls separately into a connected component. The topological (and mathematical) properties of such individual component can be analysed, compared and classified using centrality measures [47].

Specifically, we exploited the notions of cliques, communities, clustering coefficient, betweenness, articulation points, and diameter. Cliques correspond to a portion of the graph in which all the nodes are connected with one another. Communities are regions of the graphs in which all the nodes show a significantly greater proportion of connections with other nodes of the community than with any other node in the graph. The clustering coefficient of a component estimates the ratio of connections in the component over the total number of possible connections. The shortest path between any two nodes is the path of minimal length in terms of numbers of edges. The betweenness of a node quantifies how frequently this node lies on the shortest path between all pairs of nodes in the graph. Nodes with significantly high betweenness are more frequently found on these paths, and they therefore structure the network and often act like bridges. In particular, some of them are articulation points, which are single nodes that disconnect the graph into subgraphs when they are removed. Articulation points represent obligate bridges. Finally, the diameter estimates the component size: it corresponds to the largest of all shortest paths between two nodes in the component.

#### Massive tinkering in the evolution of restriction-modification endonucleases

**Figure 2:**
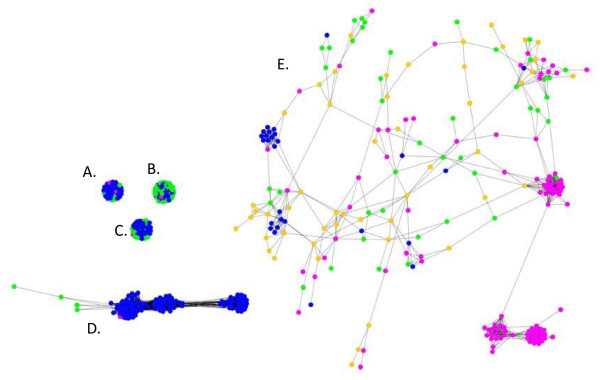
**Selected connected components of the *E. coli *gene network**. Nodes correspond to gene sequences (blue for *E. coli*, green for all other bacteria, orange for archaea, and pink for mobile genetic elements). Edges were drawn when sequences showed an homology with a BLAST score < 1e-20, > 30% identity, option false BBH. Cytoscape was used for the display. (A) Putative homoserine kinase type II. (B) Translation Initiation Factor I. (C) Predicted permeases. (D) Type V secretory pathway proteins. (E) restriction endonuclease S subunit.

For instance, we displayed the gene network (for 30% and more identity, false BBH, BLAST-score 1e-20) (Figure 2) to show that such a graph can help demonstrate that gene families under study evolved very differently. Typically, putative homoserine kinase type II, translation initiation factor I (TiF1), or predicted permeases produced very densely connected components (cliques or quasi-cliques in terms of graph theory), while restriction endonuclease S subunits genes presented a very distinct pattern of evolution, with remarkable communities (e.g. clusters of sequences) and bridges within sequences of that family. Proteins from the type V secretory pathways (adhesins, outer membrane proteins and periplasmic proteins), displayed an intermediate structure with three visible communities and showed divergent evolution as this family expanded in *E. coli*. While TiF1 and similar genes had a small diameter, typical of conserved gene families with very conserved sequences and little diversity, restriction endonuclease S subunits genes had a very large diameter that reflected a significant genetic divergence within this gene family. Likewise, the clustering coefficient (or transitivity) of these two types of families strongly differed. TiF1 genes and the like have a high clustering coefficient (close to 1), type V secretory pathways proteins have an intermediate clustering coefficient, and the restriction endonuclease S subunit family presents a much lower clustering coefficient (closer to 0). Moreover, the restriction endonuclease S subunit family presents a number of nodes with high betweenness and some local articulation points. These nodes bridge various regions of the subgraph corresponding to that family. In particular, when local articulation points are removed from a graph, the connected component is split locally into disconnected subgraphs, defining sets of rather distinct sequences within the family. Gene fusion, or domain sharing between sequences within this gene family, as well as high evolutionary rates in the family outside these central sequences, would typically result in such local articulation points [44]. Many of these nodes were sequences of mobile elements. Both nodes with high betweenness and articulation points are by contrast totally absent in the TiF1 family and similar genes, which suggests that restriction endonuclease S subunit has undergone a much more complex (non-tree-like) evolutionary history, with possibly occasional events of genetic merging or periods of strong divergence from some ancestral versions of the gene. These results are consistent with the literature [48].

Since the topological (and mathematical) properties of each individual component in such graphs can be analysed, future analyses of gene networks could therefore rely on these topological estimates to classify quickly thousands of gene families based on the topology of their subgraphs, and to automate the identification of sets of connected components (hence of gene families) with average or atypical topologies and possibly average/atypical evolutionary histories, within the framework of a gene network. This approach is particularly relevant for inferences about complex evolutionary processes. Although gene tree analyses currently benefit from a rich body of tools, which have still to be developed for gene network analyses, gene networks are more inclusive than gene trees. They are helpful not only to study LGT, but also to study the more general phenomena of transfer and recombination of genetic material. These two sources of evolutionary novelties do not always respect the boundaries of genes, when parts of genes, e.g. domains or genes fused with promoters, or when segments of DNA recombine. Such complexity is easily captured by gene networks, which allow the study of mixed evolutionary processes, which include vertical descent as well as recombination, domain fusion, etc. Moreover, the huge advantage of the gene network approach is that producing these powerfully inclusive graphs is much faster than reconstructing individual gene trees.

### A few lessons from forests

While networks are very useful and fast tools to unravel some patterns and processes of genetic diversity, they are incomparably more powerful when coupled with analyses of phylogenetic forests. The phylogenetic toolkit helps identify gene trees compatible with vertical evolution, and it allows tests of the direction of lateral gene transfer. Therefore phylogenetic analyses help determine which groups of genes were co-inherited and which were introduced by horizontal transfer before being inherited vertically. To further this objective, we present some methods for analysing patterns of genetic diversity in trees of phylogenetic forests as a valuable complement to genome and gene network analyses.

#### Clanistic analysis of the wild forest reports numerous LGTs within *E. coli*

Clanistics is a straightforward approach to analyse the evolutionary signal in a phylogenetic forest, when labels are associated to sequences under study. Sequences are first assigned to complementary categories defined *a priori *(i.e. taxonomical categories such as "*E. coli*" and "non-*E. coli*", or lifestyle categories, such as "pathogens" and "non-pathogens"). Then, clanistics proceeds by cutting trees into pieces to highlight remarkable groupings of members of these categories in the trees and in the forest. Consequently, clanistics allows the study of the dispersal of types of OTUs in the trees by partitions [42]. We used two simple partitions, clans [49] and slices to look for trees with neat groupings of *E. coli*. Clans correspond to bipartitions created by a single cut, whereas slices are obtained by two cuts of the tree [41,42]. When it is impossible to define a clean clan or a clean slice that separates *E. coli *from other OTUs, it means that sequences of *E. coli *and other OTUs are intertwined in the tree. In that latter case (mélange), non-*E. coli *sequences branch within *E. coli *sequences, either because *E. coli *transferred these genes to non-*E. coli*, or the opposite, or both if multiple exchanges of sequences belonging to this gene family occurred between *E. coli *and other OTUs. Two indices, the E* and the p-score, were used to quantify the extent of the mixing of sequences from two categories in the trees (and clans). The E* is an equitability index measuring the evenness of the distribution of sequences from a given category (e.g. all *E. coli *sequences) along the tree branches. Frequent lateral exchanges result in a positive value of the E* index (because the sequences involved in many distinct LGTs will be very mixed with that of their donor and hosts in the trees). By contrast, vertically inherited sequences will not be evenly distributed, but will all be located in the same region of the tree: perfectly grouped sequences from a given category have a null E*. The same is true for the p-scores [41]; the higher the E* index and p-scores the more frequent the mixes between *E. coli *and *non-E. coli *in the tree.

#### High rates of LGT in *E. coli*

We studied two forests: one centred on a particular *E. coli*, UTI89 (NC007946) (later called the **wild genome forest**), and another based on the genes of all *E. coli *(called the **pangenome forest**). These two forests differed in their bacterial taxonomical sampling, the former being richer in bacteria closely related to *E. coli *than the latter (see Methods). Clanistic analyses of these two forests indicated contrasting yet consistent results. The pangenome forest provided information about potential LGT above the order and class levels, and about mobile genetic elements, while the wild forest offered additional insights by accounting for both short and long distance LGTs in terms of taxonomy. Both forests indicated that mobile genetic elements seem to play a role in *E. coli *evolution. Mobile genetic elements were present in 10.3% of the wild forest (302+52+66/4065), and in 13.6% of the pangenome forest (474+184+174/6129), respectively. These slight differences reveal that a small fraction of gene families is present in the pangenome due to the impact of mobile elements, yet does not include homologues in the particular genome of *E. coli *UTI89 (NC007946). Of these mobile gene families, 28% (in the wild forest) and 43% (in the pangenome forest) had been transferred more than once between *E. coli *and the mobile genetic elements. These estimates depend on the sample of mobile elements included in the analysis, and therefore are very likely to under-represent the extent to which sequences derived from mobile elements are present in this forest, since the diversity of mobile elements is currently undersampled.

Phylogenetic proximity affected the frequency of lateral gene transfers in *E. coli*: these organisms mainly exchange genes with closely related taxa (Additional file 1A). First, analyses of the two forests showed that *E. coli *exchanged almost no genes with Archaea. These organisms may be phylogenetically too distant for successful LGT. Alternatively, the Archaea of that particular dataset may seldom share the same environments with the *E. coli *investigated here, and therefore they may not rely on the same shell genes to adapt to the environment. This interpretation would explain this low proportion of exchanges.

The pangenome forest (with no closer bacterial taxa below the order and class levels) and the wild forest (including all bacterial taxa sequenced) logically show very different estimates of LGT, due to the inclusion of closely related bacteria. The pangenome forest suggests long distance LGT (above the class level) with heavy mixing of non-*E. coli *and *E. coli *sequences in 176 trees (E*mélange = 0.7207), and perfect slices of *E. coli*, surrounded by non-*E. coli *in 186 trees. It suggests therefore that long-distance LGT affects about 5.9% (186+176/6129) of the *E. coli *pangenome. By contrast, the analysis of the wild forest, including short distance LGT (above and below the class level) returns 3174 trees (out of 4065) that exhibit heavy mixing of *E. coli *and non-*E. coli *sequences (E*mélange = 0.7362), and 343 trees with a perfect slice of *E. coli*. Thus, in the wild forest, no less than 88.9% of the trees (3174+343/4065) may have been involved in LGT events, while only 11% trees (140 + 308/4065) show no sign of LGT. These results, however, change dramatically when *Shigella *is considered as a *bona fide E. coli*. There are 1089 trees with slices and 606 trees with mélange (E*mélange = 0.55). In other words, a total of 1695 trees suggest LGT events involving *E. coli*. Still, these many trees represented a significant fraction of *E. coli *pangenome (41.7%) that seems to have been affected by LGT, and no less than 14.9% of the trees show evidence of multiple LGTs (Additional file 1A). Such a high rate of LGT is consistent with the literature [25,45].

#### Pathogenic lifestyle affects the evolution of 30% of the *E. coli *pangenome

We also used the pangenome forest to perform two clanistic analyses embracing a phenotypic perspective, focusing on the pathogenicity of *E. coli*, rather than on their taxonomy (Additional file 1B). This shift in perspective is justified, because gene exchange is very dependent on bacterial lifestyles, and because the evolution of a gene caught up in a genetic partnership will, in general, differ from that of a gene that experiences only vertical inheritance. The various strains were distinguished as pathogenic and non-pathogenic, and were sometimes associated with a specific disease (GAS, URI and HEM) (see Methods). The first clanistic analysis was achieved for all bootstrap supports, the second enforced a requirement of at least 50% of bootstrap support to resolve the strains into groups, else the non-supported branches were automatically collapsed before the analysis. This distinction based on bootstrap support had no impact on our estimates of the relative distribution of pathogenic and non-pathogenic *E. coli *in the trees. While the vast majority (70%) of the trees very strongly mixed pathogens and non-pathogens (e.g. 4291/6129 trees presented an average E* mélange of 0.9451), there was nonetheless a significant fraction of the pangenome forest that was well structured with respect to pathogenicity. 546 trees were comprised only of pathogenic OTUs, 735 trees nicely grouped all pathogenic OTUs in a perfect clan, and 547 in a perfect slice. Thus, pathogenic lifestyle affected the evolution of no less than 1828 gene families, about 30% of the *E. coli *pangenome.

When focusing on specific types of diseases, represented by smaller numbers of OTUs for three categories (URI, GAS and HEM), bootstrap support impacted the results. Therefore, we considered the clanistic results for robust phylogenies (Additional file 1B). The results regarding these diseases only yielded a limited structure in the trees of pangenome forest: 67 to 77 gene trees only cleanly grouped the taxa involved in each of these specific diseases in a perfect clan, and 367 to 680 grouped them in a perfect slice. Thus 7.2% to 12.2% of the trees showed some structure that could be related to a particular disease.

#### Detection of candidate mobile modules of pathogenicity

To further illustrate that clanistic analyses can be used to foster hypotheses about *E. coli *evolution, we also automatically identified groups of gene trees that contained mobile genetic elements (> 0 #natives when MGE are the natives), that were exclusively found in pathogenic hosts (p-score = 0 for when PATH are natives), and that presented absolutely identical taxonomical distributions in *E. coli *strains within each group. These sets of genes were likely co-inherited by lateral transfer effected by a mobile genetic element, and may be associated to pathogenicity since they are not known in any non-pathogenic organism. We obtained five groups that may correspond to five such candidate transferrable modules of pathogenicity. These candidates encoded respectively for: (i) DNA replication protein 32 and transposase and inactivated derivatives, (ii) two uncharacterized proteins conserved in bacteria and hemolysin-coregulated protein, (iii) response regulators consisting of a CheY-like receiver domain and a winged-helix DNA-binding domain, sulfite oxidase and related enzymes, and transposase and inactivated derivatives sulfite oxidase and related enzymes, (iv) signal transduction histidine kinase regulating phosphoglycerate transport system, ABC-type Fe3+ transport system periplasmic component, sugar phosphate permease, response regulator containing cheY-like receiver, AAA-type ATPase, and DNA-binding domains, and (v) predicted P-loop ATPase and predicted PP-loop ATPase.

Overall, our results indicate that an alternative approach to the TOL such as clanistics can easily sort out a forest of trees and make predictions regarding the possible implication of some gene families in pathogenicity and even specific diseases.

#### Polychromatic quartets reveal high recombination/LGT rates in core and shell genes within *E. coli*

We developed a new approach, PQ, that allows the dissection of each tree of the phylogenetic forest using quartets, by focusing on the relationships among the different strains in each and every gene tree. In a first series of analyses, all trees in the pangenome forest representing at least four different strains of *E. coli *were considered. Then, the core genes (i.e., those represented in all strains), and the shell genes (i.e., those represented in some strains only) were analysed separately to detect differences among them, if any. Finally, all trees bearing replicate (or transferred) copies of a gene in one strain were ignored to define a reduced forest of single-copy genes, which was analysed using the same PQ protocol. In short, there exist 435 (i.e., 30*29/2) pairwise comparisons among the 30 strains, and the relative frequencies of each clan appearing in PQs were tabulated in a 30 × 30 matrix. The one-complement of this matrix (e.g. a matrix with values scored as 1 - relative frequencies of each clans in PQs) was taken as an estimate of LGTs, and a splits-graph representation [50] was used to visualise any conflicts in the phylogenetic forest.

**Figure 3:**
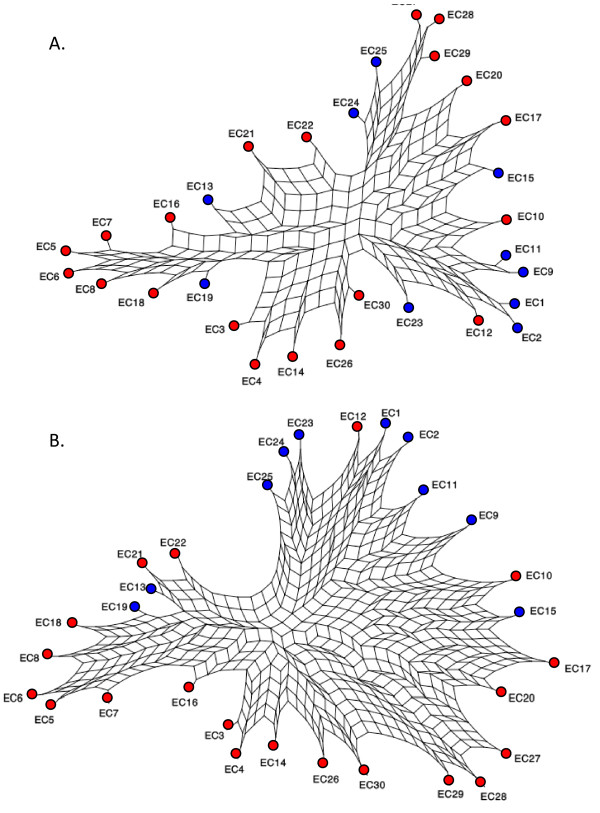
**Split decomposition graph of the *E. coli *strains**. Visual representation of the conflict in the phylogenetic signals among 30 strains of *E. coli*, for (A) the core genes (n = 2317) and (B) the shell genes (n = 3511). The strains are tagged for pathogenicity with red nodes for PATH and blue nodes for NON-PATH *E. coli*. Splitstree4 http://www.splitstree.org/ was used for both display, with the Neighbor-Net algorithm [51].

Figure 3 presents the split networks of the core (n = 2317) and the shell (n = 3511) sets of genes after a PQ analysis for the pangenome forest, constructed using the Neighbor-Net algorithm [51,52]. Neighbor-Net produces circular collections of splits depicted by a planar graph with boxes. The intricate appearance of such representations thus revealed incompatible phylogenetic signals among gene trees [53], which can be used to locate putative recombination/LGT events. If all trees had been entirely compatible, the corresponding splitsgraph would also be a tree. Figure 3 also showed that the core and shell gene sets are equally subject to recombination. A significance test (t = -36.831, p < 0.0001) indicated however that pairwise distances among strains for core genes (0.6541) are on average shorter than those for shell genes (0.8746), as also highlighted by the smaller numbers of "reticulate" cells in the corresponding networks. This observation suggests that core genes are less likely to be transferred than shell genes, in regard to the pangenome forest we have analysed.

#### Preferential exchanges of DNA material between pathogenic *E. coli*

To assess the effect of pathogenicity on recombination/LGT frequencies, the different strains of *E. coli *were tagged as PATH (n = 20) and NON-PATH (n = 10) (e.g., Figure 3, red and blue nodes). A Mantel test [54] comparing the distances among the different strains with respect to pathogenicity was used to assess whether distances among groups (PATH vs NON-PATH) were significantly larger than those within groups (PATH vs. PATH and NON-PATH vs. NON-PATH). The results revealed that a pathogenic strain is more likely to exchange genes with another pathogenic strain than with any other non-pathogenic strain, for the whole set of genes (r = 0.1511, p = 0.024), the single-copy genes (r = 0.1380, p = 0.035), and the shell genes (r = 0.1815, p = 0.015), but not for the core genes (r = 1215, p = 0.1), which barely miss the significance level for multiple tests. This result can be explained due to the ability of pathological species to meet in the gut, which would enhance their rate of LGT. It confirms that the LGT of shell genes is likely to have adaptive effects, i.e. related to ecological/lifestyle phenotypes, and that the LGTs are possibly selected for, since we observed here an increased rate of LGT/recombination for shell genes between pathogens, distinct from the background rate of LGT/recombination of core genes that cannot make an ecological/lifestyle difference in *E. coli *hosts.

All computations were performed with a cutoff level of 50% bootstrap for including a polychromatic quartet in the analysis, but qualitatively similar results were obtained for other bootstrap values, and even without taking bootstrap support into consideration (results not shown). There were not enough data available for PQ to detect any preferential LGTs among strains of *E. coli *causing the same types of diseases (URI, GAS or HEM).

Overall, application of the PQ approach revealed complex and intricate phylogenetic patterns among the different strains of *E. coli*, and the importance of pathogenicity for LGTs. Whereas the clanistic methodology can help define homogeneous groups of OTUs (clans and slices) by focusing on bipartitions and tripartitions, PQ looks for significant patterns at a different scale, by dissecting trees in quartets of leaves. Consequently, this is the locus of the statistical power of this approach, which allows specific evolutionary hypotheses to be tested by colouring the leaves using diverse categories (i.e., the different strains, pathogenicity, diseases, etc.), while clanistics methods, accounting for two categories (X vs. non-X) are still restricted to statistics on bicolour trees.

## Conclusions

Our present goal was certainly not to offer a new detailed picture of *E. coli *evolution, even though we could confirm many well known facts about the prevalence of recombination and LGT in *E. coli *[25,45] , and support some new hypotheses (e.g. suggesting gene families and gene modules involved in pathogenicity, pointing out strong evidence for recent LGT as exemplified by large numbers of transferred genes involved in replication and repair, etc.). The set of analyses deployed here had only one purpose: to illustrate that there exist alternative ways to study evolution beyond the TOL research program. The diversity of processes and elements that can be included in evolutionary scenarios (genes, genomes, functions, mobile genetic elements, cellular organisms, pangenomes, genetic partners, etc.) strongly suggests that no single approach could provide an exhaustive description of microbial evolution. Therefore, depicting a unique picture of evolution (whether a web or a tree) may not be the only future option for evolutionists. Rather, the use of multiple distinct tree-based, forest-based and network-based approaches may be a more powerful way to characterize the evolutionary processes and mechanisms that sustain diversity, even within a well-defined microbial group.

The TOL is one of these many possible approaches to decipher evolution; therefore it is one of many possible heuristic ways to deal with understanding natural diversity and its history. If our simple case studies motivate more evolutionists to explore a wider range of methods beyond the TOL, i.e. to explore woods and webs, for which conceptual and methodological developments are still in their infancy, rather than defaulting to a single practice, this paper will have achieved its goal. Many open questions, not addressed by the TOL, will indeed require the focus of evolutionists. For example, as suggested by Richard Burian, it might be timely (i) to explore the variation in the rates of lateral transfer in different gene families, and (ii) to devise ways to determine whether there are differences in selection regimes when genes from a given family are embedded in viral or plasmidial genomes on the one hand, or in cellular genomes on the other hand. If indeed genes that undergo LGT experience independent evolutionary processes (e.g. different selection regimes) when they reside in mobile elements than while they reside in cellular genomes, novel models of molecular evolution, beyond the TOL, will be required. More generally, the necessity of including mobile elements in the evolutionary picture along with the cellular chromosomes is now raised. Similarly, as pointed by Didier Raoult, (iii) future work will need to make room for ORFans. These sequences will pose additional methodological and conceptual challenges for evolutionary studies, since comparative approaches are not designed to handle unique sequences that cannot be compared to any other sequences. Such efforts to go beyond the TOL indeed support the recognition of the extraordinary complexity of evolution: methodological pluralism is an important step toward its understanding.

## Methods

### Reconstruction of the dataset

We downloaded all chromosomally-encoded proteins (141,493 sequences) for 30 strains of *E. coli *from the NCBI ftp site, carefully excluding protein sequences encoded by plasmids. The list of strains and their lifestyles are detailed in Additional file 2. These proteins were added to a pre-existing database of 300,841 proteins from 119 cellular genomes and 22,131 sequences of phages and plasmids, obtained from the NCBI. Gene families were reconstructed as follows, consistent with procedures in [21,43]. First, each sequence of this extended dataset was compared against one another by reciprocal BLASTs (1e-5 cutoff). Second, gene families were defined by clustering homologous sequences using a single-linkage algorithm. This method grouped sequences if they shared a reciprocal best-BLAST hit relationship with at least one of the sequences of the cluster (option "true BBH"), or simply if sequences presented a minimal homology score of 1e-20 (option "false BBH"). Third, for phylogenetic analyses of the pangenome forest (see below), an additional criterion was enforced: sequences were clustered in a same family by the single-linkage algorithm (false BBH) if reciprocal BLAST hit pairs shared a minimum sequence identity of 70%. For network analyses, various identity thresholds were used in the analysis: [41], [90-99%], and [100%] were used to obtain distinct gene and genome networks.

### Sequences annotations

Each sequence of the dataset was functionally annotated using RPS-BLAST [55] with a COG profile database. Each of the sequences investigated was also labeled according (i) to its host type (MGE for mobile genetic element, e.g. Virus + Plasmid; EUK for Eukaryote; ARC for Archaea; BAC for Bacteria; and EC1 to EC30 for the different strains of *E. coli*), and (ii) with respect to the available information on its host pathogenicity (NON-PATH for non-pathogenic hosts, PATH for pathogenic hosts, OTH when this information was unknown), through a careful inspection of the organismal annotation of the GOLD table [56]. Sequences from pathogenic *E. coli *only were then further tagged according to the type of disease they were causing: URI for urinary infection and cystitis, HEM for hemorrhagic colitis, GAS for gastroenteritis, OTH for other diseases. These annotations were further used in network and forest analyses.

### Phylogenetic analyses

We used the entire genome of *E. coli *UTI89 (NC007946) as a seed, and BLASTed all its 5021 genes against the nr database (from the NCBI) to produce a phylogenetic forest centred on *E. coli *UTI89. Each gene was aligned with all its homologues with a BLAST score > 1e-5 using MUSCLE [57]. Ambiguously aligned regions were excluded using GBlocks [58], which let us with 4065 unambigously aligned families with over 3 OTUs, for which phylogenetic trees were inferred by ML using Phyml [59] (WAG model, empirical character frequencies, estimated invariant proportion). The sequences in these trees were also automatically annotated as MGE for mobile genetic element (e.g. Virus + Plasmid), EUK for Eukaryote, ARC for Archaea, BAC for Bacteria, and EC1 to EC30 for the different strains of *E. coli*. This first forest, referred to as *E. coli *UTI89 wild forest, was used to investigate the amount of LGT between *E. coli *and all sorts of relatives (i.e. from closely related bacterial species and genera to OTUs of other Orders and Families, Domains).

We also constructed a second forest, the *E. coli *pangenome forest, sampling a greater number of *E. coli *genes but for a different diversity of prokaryotic lineages, as described above. Since only three OTUs belonged to the same order as *E. coli *in this pangenome dataset (*Coxiella burnetii *RSA 493, *Psychrobacter arcticus *273-4, *Shewanella putrefaciens *CN-32), this second forest can only investigate the evolution of the *E. coli *pangenome at two levels: the recombination/LGT between *E. coli *strains and the LGT between *E. coli *and distantly related OTUs (e.g. LGT above the order and class levels). Gene families (false BBH, > 70% identity) without any *E. coli *sequences were excluded from the analyses: 7726 gene families with at least one *E. coli *were selected to reconstruct *E. coli *phylogenetic forest at > 70% identity. Each family was aligned with MUSCLE and GBlocks as indicated before, and trees inferred by Phyml v3.0 (same options as above). For each gene tree in the pangenome forest, 100 bootstrap replicates were performed with the same parameters. This analysis resulted in 6129 individual trees with at least one *E. coli*.

### Network analysis

Gene and genome networks were reconstructed as in [21,43], respectively, for the gene families defined above. We used pre-implemented centralities of Igraph R package (betweenness, diameter, degree, articulation points), and in-house Perl scripts (available upon request from CB and PL) to analyse *E. coli *gene and genome networks.

### Clanistic analysis of the forest

*E. coli *wild and pangenome phylogenetic forests were analysed with an updated version of the getDiversity function of the Phangorn R package [41] to identify perfect (trivial and non-trivial): clans, slices, and to compute intruder indices. Scripts achieving these analyses are available upon request from KS. Candidate mobile modules of pathogenicity genes were obtained through a critical selection of gene sets based on two covariables: MGE, and PATH. Namely, trees with similar distributions of taxa with a number of MGE > 0 and a p-score = 0 for PATH = natives were sorted out with an automated R script, identifying groups of mobile genes with identical yet odd taxonomical distributions of pathogens.

### Implementation of the polychromatic quartet (PQ) approach

The polychromatic quartet approach was applied to detect a mélange among some *E. coli *strains at a finer scale than the tree, using a new function implemented in R. To do so, each tree of the forest was analysed by (1) sampling at random a quartet of *E. coli*, and (2) coloring the leaves with respect to the four different strains; e.g. blue (B), red (R), yellow (Y), green (G). Out of the three possible unrooted topologies for four OTUs, (3) the bipartition supported by the data was selected (e.g., BR|YG). (4) The corresponding clans (e.g., BR and YG) on both sides of the bipartition were tallied. (5) This process was repeated for 1000 quartets to compute occurrences of all clans in the polychromatic quartets. (6) A 30 × 30 pairwise matrix was assembled by combining the results for all gene trees, and (7) further analysed with the Neighbor-Net [51,52] algorithm in Splitstree4 [50,60] to depict the relationships among the different strains and reveal any conflicting signals in the forest. The PQ approach was performed on the entire set of gene trees (5828 trees with at least four OTUs), as well as for a set of core genes (2317 trees bearing the 30 strains), a set of shell genes (3511 trees bearing less than 30 strains), and a set of single-copy genes (5018 trees bearing no more than one copy of the gene for all strains). The analyses were performed while taking into account boostrap support (> 50%) to obtain robust results. Host pathogenicity (and diseases) were then used to test whether some strains, or some sets of genes, were more likely to be subject to LGTs among particular categories (core genes vs. shell genes, pathogens vs. non-pathogens).

## Abbreviations

ARC: Archaea; BAC: Bacteria; BBH: Bidirectional Best Hit; BLAST: Basic Local Alignment Search Tool; COG: Cluster of Orthologous Genes; DNA: Deoxyribonucleic Acid; EC: *E. coli*; EUK: Eukaryote; FOL: Forest of Life; GAS: Gastroenteritis; HEM: hemorrhagic colitis; LGT: Lateral Gene Transfer; MGE: Mobile Genetic Element; ML: Maximum Likelihood; NCBI: National Center for Biotechnology Information; NON-PATH: Non-Pathogenic; OTH: Other; OTU: Operational Taxonomic Unit; PATH: Pathogenic; PQ: Polychromatic Quartet; RNA: Ribonucleic Acid; TOL: Tree of Life; URI: Urinary infection.

## Competing interests

The authors declare that they have no competing interests.

## Authors' contributions

EB and FJL conceived the study and wrote the manuscript. All authors participated in data analyses, and read and approved the final manuscript.

## Reviewers' comments

### Reviewer report 1 by W. Ford Doolittle (Dalhousie University, Canada)

I have nothing useful to say about the individual methods presented by Beauregard-Racine and colleagues, but one extended comment on the pluralistic approach they together embody. It is worth reminding ourselves that there is very little difference between the various sides in the TOL debate in terms of understanding of the genetic and ecological processes that determine the structures of individual genomes or the evolution of individual genes. There is not even much disagreement about the relative extents of verifiable vertical descent and LGT. What we are arguing about are relative importances and appropriate representations, matters of generalization about which there may be no facts. All that's really out there in the world are these genetic and ecological processes affecting and having affected one gene or one organism at a time over four billion years. So the pluralism endorsed in this contribution may not only be more useful (in suggesting new ways to look for new things), but more true, in that it discourages us from seeking generalizations and thinking of them as laws.

*Authors' response: We fully agree with Ford Doolittle, and thank him very much for his major role in extending the research field of evolutionary biology beyond the TOL*.

### Reviewer report 2 by Tal Pupko (Tel-Aviv University, Israel)

In bacterial evolution, the hypothesis of "one tree to rule them all" is now widely rejected. In other words, there is not a single species tree topology that describes the evolution of all the genes - different gene trees have different topologies. Those different topologies cannot be explained by stochastic noise or phylogenetic artifacts. The lack of one true tree immediately calls for networks as a visualization and analysis tool to study bacterial evolution, be it either a genome network or gene network. In this paper, Eric Bapteste and colleagues clearly explain the need for networks to study bacterial evolution; they survey some network methodologies and apply them to study the genome evolution of *E. coli*. The paper provides easy exposition to these network tools, and how they can quickly be used to visualize evolutionary dynamics. Given the ever increasing number of bacterial species for which dozens of isolates have their genomic sequences fully determined, the utility of such methods is expected to increase significantly.

Since this is more of a review paper than a research paper, I would have liked to see more discussion about the open questions in the field (computational and biological challenges in the field of network analysis). Furthermore, many of these network analyses provide results that can also be obtained by other methods. I think it is important to mention other methodologies that aim to answer the same questions as those provided by network-based analyses. As a case in point, maximum-likelihood analyses of gene family presence and absence (phyletic pattern analyses) have provided many insights into genomic fluidity within and among bacterial species.

To summarize, this nicely written work clearly demonstrates the need for novel methodologies to analyse bacterial genome dynamics, methods that differ from those used to analyse the TOL. I expect that as more data accumulate, Bayesian and likelihood based inference tools will be used to capture better the peculiar evolutionary processes that cause genome fluidity in bacteria. This paper and others also seem to indicate that the involvement of phages in bacterial fluidity is underestimated and that bacterial genomics is tightly linked to molecular biology and evolution of phages.

*Authors' response: We thank the referee very much for his comments. He is absolutely right on all grounds. There are indeed many open questions in the field of network analyses, but this particular issue would certainly deserve to be the focus of a separate paper. In this revised version, we mention some biological open questions associated with network approaches. However, we fully share the referee's interest, and we would like to encourage motivated colleagues to elaborate reviews on the computational and biological challenges in the field of evolutionary network analysis. Some good leads for this useful and timely work could for a start be found in the excellent special issue of 2009: *[12,61]. *As methodological pluralists, we can only welcome the development of novel methods (based on maximum likelihood, Bayesian analyses, and specifically accounting for gene family presence and absence)*.

### Reviewer report 3 by Richard M. Burian (Virginia Tech, USA)

During the last half-dozen years of so, Eric Bapteste and numerous colleagues have developed a long-term program of research aimed at providing a pluralistic framework for interpreting (mainly prokaryotic) processes of genomic change and evolutionary patterns in terms of networks of exchanges among genetic units of various sorts. The present manuscript explores lessons that can be gleaned from applying four different methods, two of them network methods, two of them methods for analysing the "forest of life" (FOL), i.e., the forest of (divergent) gene trees, employed on genomic and genetic data for *E. coli *and various archaea, bacteria, and mobile elements (plasmids and phages). A major purpose of the submission is to show how the application of different methods to large datasets can handle a diverse range of questions by following a variety of evolutionary units that evolve on different scales and in different patterns. In particular, real data in the highly fluid pangenome of *E. coli *serve as a model for application of this set of tools and methods to capture different sorts of units and different rates and kinds of exchanges that are more helpfully analysed via network and FOL tools than with standard tree-based analyses. The methods applied to the FOL utilize the concepts of *clans *(created by bipartition of trees of operational taxonomic units, often unrooted,) and *slices *(segments between two cuts in such unrooted trees). These methods provide evidence of lateral gene transfer into and/or out of clans or a slices; analysis of such transfers proves to be of considerable importance. In addition, a novel method analysing "polychromatic quartets" (involving pairwise comparison of gene trees that contain at least four distinct strains, here, with data for 30 strains of *E. coli*) allows a finer-grained analysis of lateral transfer. In the *E. coli *data, this tool was able to demonstrate, for example the (possibly surprising) result that (except perhaps for genes in the *E. coli *core) lateral exchange among pathogenic strains of *E. coli *has occurred more frequently than between pathogenic and non-pathogenic, or among non-pathogenic strains.

As a philosopher of biology who is not equipped to evaluate the methods as such, I concentrate on the results rather than the methods. The results of greatest interest concern the evidence for the extraordinary degree of genetic mosaicism both in recently evolved taxa and in the long-term evolution (and co-evolution) of a wide range of bacteria, archaea, and mobile elements.

To my eye, what is most striking is the fine tuning of adaptation achieved by lateral transfer, which, for archaea, bacteria, and mobile elements, serves something like the role of recombination in eukaryotes. Of particular interest is what this sort of work suggests regarding debates over the units of evolution. The perspective of the authors is firmly pluralist: they view their tools as exploratory, pragmatically accepting as units whatever entities the data show to have relative autonomy over a relevant range of variation within or among a relevant range of genomes. In short, they claim to utilize the data to identify, locate, and pursue different units of evolution, operating on different scales and in different contexts without strong advance commitments about the full-fledged autonomy of the units or the topology of the trees or networks within which they are found. In general, their findings, as I understand them, suggest that both the structure and the selective values of all units of evolution depend on context, including the other units of evolution with which they interact and (for genes and other embedded sequences of DNA) which sorts of entities they are embedded in. Given LGT, there is both intergenic and intragenic recombination across (larger) evolutionary units. The recombination does not respect the standard phylogenetic boundaries; exchanges take place among archaea, bacteria, and mobile elements, though, of course, at widely different rates. Such findings provide empirical support for a pluralist position, according to which the status of units as (locally and functionally) fundamental depends on the contexts considered and the scale of investigation (e.g., the genomic contexts of the units, the processes by which exchange occurs, the relative stabilities of the units among which there is evolutionary competition, and the extent of the environmental and organismal interactions under investigation).

The conceptual issues of greatest interest concern the extent of the effects of "genetic partnerships" between, e.g., mobile elements and cellular genomes, or across cellular genomes. Such entities as "mobile modules of pathogenicity" can be uncovered by the investigative methods developed by the authors (and others) and appear unlikely to be well understood without understanding the lateral transfers that are involved. More generally, the ways in which the units uncovered depend on the questions investigated, the scale of changes examined, and the investigative tools employed, strongly suggest that a pragmatic and pluralist understanding of the units of evolution and of genetic function is appropriate to the ongoing stream of investigations of evolutionary patterns and processes.

This general characterization provides the interpretative framework that I understand (from the present submission and from some previous publications) the authors to employ. I find little to criticize in the general framework, but have some questions at a finer grain. I address these questions directly to the authors.

*Authors' response: We thank the referee: he described with very much insight the logic of our (past and present) contributions. It is a real honour from such a great specialist of history and philosophy of biology*.

In the abstract, you mention *genetic partnerships *twice, but that concept never appears directly in the text of the article. It might help to revisit it in some fashion later in this paper, for the evolution of a gene caught up in a genetic partnership will, in general, differ from that of a gene that experiences only vertical inheritance and/or no effects from a symbiotic relationship.

Authors' response: We agree and have added this claim into the revised MS: "the evolution of a gene caught up in a genetic partnership will, in general, differ from that of a gene that experiences only vertical inheritance"

Similarly, although you are clear that methodological pluralism is called for in dealing with different (evolutionary) questions, it is not clear whether you wish to take a strong position about the extent to which the boundaries of evolutionary units drawn or accepted by investigators depend on the questions they are pursuing and the investigative tools that they use. This may not be the appropriate place to address that issue, but it is one that needs to be addressed carefully at some point in following up the lines you have opened up here and elsewhere. Does it deserve a comment in the present context?

*Authors' response: Indeed, we wish to take that strong position: the boundaries of evolutionary units we draw depend on our questions and tools. There are so many connections in an evolutionary network, so many interactions and types of interactions, that results of scientific inquiries looking for some structure in this evolutionary web will always stress some privileged connections, for pragmatic and instrumental reasons. However we (evolutionary biologists) will particularly value the boundaries (and relationships) grounded in a biological process: our tools and questions can also be designed to try to unravel evolutionary groups based on evolutionary processes. By analogy, these groups can be seen as the consequences of "questions" asked not only by investigators, but also "asked" to the evolving entities by their biotic and abiotic environments (i.e. how to survive in a hypersaline environment with reduced organismal diversity, how to survive in an arms race with a predator, etc), defining some boundaries (e.g. in the sharing of some traits) and introducing some structure to the evolutionary web. When the investigators' questions can be framed in terms of "natural selection" for example, the units identified are easier to interpret and explain in an evolutionary framework, even without a TOL. Some researchers may therefore be willing to attribute a stronger ontological reality to these remarkable units (and their remarkable connections) than to consider them merely as conventional (pragmatically-defined) objects (which of course they are as well). Such units would be in some respect "hard" conventional objects (as opposed to "soft" conventional objects, purely stemming from the focus and interest of human minds): such units would still impact and emerge from the ecological and genetic processes mentioned by Ford Doolittle, even if no human investigators was around to study them. They would constitute aspects of biological reality with their own local causal effects. We would be interested to hear whether this intuitive (likely naïve) philosophy on units seems sound to the referee, and how it could be improved (or replaced)*.

You claim in the second paragraph of the *Background *that homologous characters comparable across all life forms are needed in order to reconstruct the TOL. I'm not convinced that this is correct. If there are several major evolutionary transitions (e.g., from a pre-DNA to a DNA-based genetic system, etc.), there may be no reason to expect ANY character to be identical by descent with a sufficiently distant ancestral character. If homology means something approximating identity by descent, your claim seems to require too much of those who seek to reconstruct a single TOL.

*Authors' response: The referee is right. If there are several major evolutionary transitions, homology might not be a sufficient guideline to describe early evolution. For such a difficult task, this central notion must be complemented (or replaced) by additional evolutionary concepts. We edited the text accordingly*.

In the fourth paragraph of this section, you might want to make a clearer (or stronger?) claim about the difficulty affecting inferences from pattern to process caused by the independent processes impacting the evolutionary histories of genes. This seems crucial both for the support of your pluralism and for your emphasis on the need to work on the impact of multiple processes on pattern in evaluating inferences from pattern to process.

Authors' response: This is a crucial point that certainly justifies pluralism in evolution. Evolutionary patterns (most obviously the most complex ones, i.e. phylogenetic networks) are indeed caused by independent processes impacting the evolutionary histories of genes. From a pluralistic perspective, methods specifically designed to tackle this issue (e.g. that there is often more than one process behind a pattern) must be encouraged, as opposed to attempts to explain all patterns by a single process (e.g. all evolution by a tree-like process of descent). We clarified this in the revised version of the manuscript, see the section "This kind of phylogenetic networks put forward [...] A tree alone is not going to help establish much of this evolutionary complexity."

In the second paragraph of the *Results and Discussion*, you claim to divide gene networks into temporal slices. Strictly speaking, this seems to be incorrect. As you indicate in a parenthetical comment, 100% identity of certain sequences in the data for the genome of an *E. coli *strain and a mobile element might be caused by recent exchange or by very strong purifying selection. It *is *plausible that the data for the 199 mobile elements and the various *E. coli *strains you examined do not result from purifying selection, but the claim that the data provide temporal slices is the conclusion of an argument, not appropriate as an initial characterization of the slices themselves.

*Authors' response: We agree. We removed "temporal" before slices, and only concluded afterwards that the slices we studied at 100% identity treshold were likely to correspond to recent events of sharing*.

Minor query: In the next paragraph, you report that Table 1 shows 41% of the 4361 100%-similarity sequences belong to the L functional category another 41% belong to the unknown function category. In working through the table to be sure that I understood your results, I found that (1838/4361) = 42.2% and (1832/4361) = 42.0%. So either I misunderstood the calculation or the numbers should read 42%.

*Authors' response: Sorry, we fixed that number to 42%*.

In paragraph 4, it might be worth adding a sentence or two (if it is correct) to the effect that your analysis suggests that gene networks are more helpful than gene trees in producing plausible inferences from evolutionary patterns to evolutionary processes - at least where lateral transfer is involved and leaves traces that have not yet been erased.

*Authors' response: It is to some extent correct, although currently phylogenetics benefits from its history of use and from a rich body of tools to study gene trees, all of which would still need to be developed for gene networks. Yet, gene networks can be seen as more helpful than gene trees for inferences on complex evolutionary processes, since they are more inclusive than gene trees, and allow the investigation of mixed evolutionary processes that included vertical descent as well as recombination, domain fusion, etc. However, gene networks are not polarized like gene trees are, and they harbour no nodes corresponding to hypothetical ancestors. Future developments are likely to produce some improvements on these fronts. We have added a quick sentence in the text to introduce these claims*.

In the section on *lessons from networks*, as part of the discussion of the results, it might be useful (if you think it correct) to suggest that the genes that exhibit LGT (including the ones that hitchhike with replication and repair genes) may well experience independent evolutionary processes (e.g. different selection regimes) while they reside in mobile elements than while they reside in cellular genomes. This exemplifies, as I understand it, a key reason for which direct inference from pattern (in trees) to process is fragile. If you agree, perhaps this would fit best into the last paragraph of this subsection.

*Authors' response: We agree entirely. This may very well be an important distinction, worth modeling, that is currently missing in methods trying to reconstruct the TOL, as these mobile elements, or the trajectory of genes in and out these elements coupled to possible changes in selection regimes, is not modeled in TOL-based approaches. This issue calls for the inclusion of the mobile elements, and their selection regimes, in models of molecular evolution. We have briefly discussed this topic in the revised manuscript*.

In the *Lessons from the Forest*, first paragraph of the section on *Clanistic analysis*, it would help if the E* index is explained. I have only a first approximation understanding of this index, but it seems unlikely to me that it can serve as a wholly general way of distinguishing intruders from natives in the intended sense. It is, or should be, an empirical question whether sequence partitions into clans and slices present so extensive a mélange that (in some cases) no clear answer derived simply from the sequence data as to what should count as a native is available. Abstractly, at least, insofar as the E* index is concerned, this seems to be an open question, though one that (I suspect) the data will resolve favorably for most of the familiar sorts of cases that have been examined. But as more esoteric sorts of genetic units and more difficult sorts of genetic partnerships are explored, there may be some surprises on this front. In any case, some sort of explanation, if feasible in brief compass, of the E* index would be of use.

Authors' response: The referee is right. It is indeed an empirical question whether the partition in clans or slices will show extensive mélanges of two categories of OTUs. The E* quantifies the extent of this mixing between entities belonging to two categories defined a priori. These categories are for now arbitrarily defined, rather than inferred from the data. Although they are currently called "natives" and "intruders" but they could very well have been called "cat1" and "non-cat1". We have added a brief explanation of the E* in the revised version of the MS.

In the next paragraph, what exactly do you mean by the claim that "Mobile genetic elements were present in 10.3% of the wild forest"? My assumption is that in 10.3% of the gene trees in the database, sequences matching some sequence in the *sample *of mobile elements included in the analysis were present. If that is correct, this result is likely to underrepresent the extent to which sequences derived from mobile elements are present in this set of trees. If it is incorrect, you need to clarify what your claim means. The importance of the sample in determining the fraction of gene families that have been impacted by mobile elements is unclear, but one might suspect that the number of gene families showing such impact might increase as we explore other wise of identifying sequences that have been impacted by LGT.

*Authors' response: The referee's first interpretation is correct: the 10.3% depends on the sample of mobile elements included in the analysis, and therefore are very likely to underrepresent the extent to which sequences derived from mobile elements are present in this set of trees, since the diversity of mobile elements is currently undersampled. We have made this point clearer in the revised MS*.

The conclusions do a nice job of summarizing important aspects of the findings of this paper and putting them into perspective. They might perhaps be expanded with a sentence or two about further steps suggested by the material reported on in this paper and/or by the general approach of the group that have contributed to this line of research. For example, two general directions that stand out for me are (1) exploring the variation in the *rates *of lateral transfer in different gene families (and, perhaps, the need to devise methods to detect lateral transfer in those gene families where such transfers are very rare) and (2) devising ways to determine whether there are differences in selection pressures or the direction of evolution (e.g., in GC content) when genes from a given family are embedded in viral or plasmidial genomes on the one hand, or in cellular genomes on the other hand.

*Authors' response: These open questions are indeed important ones; we have introduced them in the revised MS*.

### Reviewer report 4 by James McInerney (Maynooth University, Ireland)

This manuscript deals with a few different issues relating to how prokaryotic genomes evolve. Of significant interest to many scientists are the methodological developments and the Polychromatic Quartets approach to the analysis of genome fluidity is indeed quite interesting. I have very few issues that I wish to raise and I think this is a useful addition to the literature in this area.

*Authors' response: We thank the referee for his comments*.

On page 6 in the last paragraph, you say that "[...] these genome networks highlighted that *E. coli *shared 90-100% identical genes with two pathogenic genomes [...]". Does this mean that it shares -**some**- sequences that are 90-100% similar? I think this is what it means, but I think this could be clarified a little.

*Authors' response: Yes, we clarified this*.

Of interest in the group of genes listed as being common to *E. coli *and *Acholeplasma laidlawii *is a 30S ribosomal protein S12. This is a slowly evolving gene and so perhaps it is shared through vertical rather than horizontal transfer. Are there any phylogenetic trees suggesting that there is a specific sister-group relationship between *E. coli *and *A. laidlawii*?

*Authors' response: In fact, it is E. coli and S. putrefaciens that share the 30S ribosomal protein S12. They are both gamma-proteobacteria. In our dataset, if this sharing was only due to vertical descent, two other taxa, also closely related to E. coli (Coxiella burnetii RSA 493 and Psychrobacter arcticus 273-*4) *may have shared this rps12. We can certainly not rule out that this particular connection for rps12 reflects vertical descent however*.

*Concerning E. coli and Acholeplasma laidlawii: they are not closely related. Acholeplasma laidlawii is a mollicute. Interestingly, it is known to produce extracellular vesicles packaging genetic material *[62]. *As this process of vesiculation, generally captures random DNA found in a host cell, the shared transposase could very well have been transferred by this mechanisms*.

Page 8: "The phylogenetic framework helps identifying gene trees compatible with a vertical evolution [...]" needs to be changed

*Authors' response: We changed the sentence*.

Page 8: "Either some non-*E. coli *branch within *E. coli*: [...]" You probably need to say "Either some non-*E. coli *-**sequences**- branch within *E. coli *[...]"

*Authors' response: Yes, we edited the text accordingly*.

Page 8: This sentence needs to be clarified: "First, analyses of the two forests showed that E. coli exchanged almost no genes with Archaea that appeared too distantly related."

Authors' response: We clarified the sentence. The revised version reads: "First, analyses of the two forests showed that E. coli exchanged almost no genes with Archaea. These organisms may be phylogenetically too distant for successful LGT. Alternatively, the Archaea of that particular dataset may seldom share the same environments with the E. coli investigated here, and therefore they may not rely on the same shell genes to adapt to the environment. This interpretation would explain this low proportion of exchanges."

Page 10: "The one-complement [...]". Could you say briefly what the one-complement is?

*Authors' response: The one-complement corresponds to matrices in which values comprised between 0 and 1 (relative frequencies of each clans appearing in PQs) have been substracted from 1*.

There are quite a few typographical errors and these should be sorted-out before publication - I don't wish to go through each of them one by one.

*Authors' response: We edited the article carefully*.

### Reviewer report 5 by Didier Raoult (La Timone, France)

Thank you for giving the opportunity to review this paper which emerges at the time when the theory of the TOL becomes increasingly unstable, and does not appear likely any more to be really defended. This analysis of the pangenome stimulates some reflections. I think that the integration of these elements could bring to have a more ecological vision which could enrich the discussion.

Authors' response: We thank the referee very much. We agree with his views: a more ecological vision could enrich evolutionary studies beyond the TOL. To strengthen this claim, we now explain in the revised manuscript that: "This realization had some impact on phylogenetics, which had historically considered evolution through the lens of systematics rather than ecology. Core genes, often assumed to be vertically inherited, were typically expected to produce a fundamental vertical framework, against which the evolution of traits and lineages was to be interpreted. Such core genes appeared suited to think about "groups within groups", which is a logic consistent with systematics. However, the distribution of shell genes was clearly explained by additional evolutionary processes, involving in particular gene transfers between partners with overlapping lifestyles or environments. Most of gene evolution (that of shell genes) appeared therefore better interpreted in light of an ecological vision."

1. Regarding the exchange of genes, this is very dependent on the lifestyle of the bacteria. Bacteria exchange genes when they live together, and when the species are sympatric. We recently proposed the use of this definition to differentiate the bacteria which live isolated in an ecosystem (allopatric) to those which live in complex systems comprising many species (sympatric) by transfer of the concept of Mayr. Concerning human *Escherichia coli*, which has been much studied, they live in complex communities in the digestive tract; a very recent paper [46] shows that the bacteriophage population in the digestive tract is huge, explaining why in this ecosystem the bacterial species exchange many genes because a very significant number of phages and generalized transduction. This basic finding appears very important to me to explain these major genomic repertoire changes [63,64].

*Authors' response: We agree. We now stress more strongly that gene exchange is very dependent on bacterial lifestyles, and we have included in the manuscript the reference to bacteriophage populations in the gut *[46], *since we now report that our results are "consistent with previous findings *[46], *highlighting the role of huge viral populations to provide adaptive genes to their cellular hosts in the digestive tract"*.

2. A second point that could be developed is the impossibility in a certain number of cases of making trees of genes because of the importance of recombination. A recent work published on *Legionella *shows that sympatric bacteria recombination reaches a huge level that appears more related to genetic and ecological proximity than to any other factor [65]. This reinforces the idea that sympatric bacteria are all recent mosaics of gene sequences. In addition the recombination introduces the idea that term LGT is inappropriate and should be replaced by LST for Lateral Sequence Transfer. The idea of LGT is a functionalist idea which does not have any meaning, since it is only selective purification that is functionalist. The transfer is mechanical and does not have a goal (Court Jester theory). However this confirms well that the phylogenic proximity is one of the elements allowing easy recombination and the lateral transfer of sequence.

*Authors' response: Two really good points. It is absolutely true that in certain cases gene trees do not reflect gene evolution (i.e. due to recombination, domains fusions, unequal evolutionary rates affecting homology detection and excluding fast evolving sequences from phylogenetic alignments). For those very likely common cases, other representations than trees may be better suited to study evolution. It is precisely for that reason that we have started developing gene networks*.

*It is also absolutely true that what transfers is genetic material (DNA or RNA sequences). Thus LGT is a particular case of LST, when the DNA fragment that was transferred functions as a gene. Some sequences functios as genes in multiple genomic contexts, whereas others don't. Gene networks are thus really good tools to study both recombination and LST. We have discussed and clarified these two points in the main text*.

A point which appears to me to be an object for future work is to integrate the most pathogenic *Escherichia coli: *that is, *Shigella*. *Shigella *are among *Escherichia coli *phylogenetically but they present an extremely reduced genome because of their strict dependence on the host in contrast to *Escherichia coli*. Pathogenic *E. coli *do not have a degree of evolution in the pathogenicity, comparable at those of *Shigella *[63].

*Escherichia coli *remains a very large pangenome but we have a bias of selection because non human *Escherichia coli *are not yet sequenced at the same level. It appears that the most important source of *Escherichia coli *is animal (poultry, pigs, etc). The level of exchange between pathological species is probably also related to the fact that they have the capacity to meet in the gut, which is more important than with the non-pathogenic species. Finally beside the core genes of shell genes the authors do not analysed the ORFans, which represent the creativity of bacteria. It would be interesting to have at least an idea of the proportion of ORFans in each isolate from the pangenome, in order to have an idea of their proportion.

*Authors' response: We have added the notion that pathological species may be able to meet in the gut, which would enhance their rate of LGT. The referee is also absolutely correct thatfuture works, beyond the TOL, will need to make real room for ORFans. These sequences pose a great methodological and conceptual challenge for evolutionary studies since comparative approaches are not in the first instance designed to deal with unique sequences that cannot be compared to any other sequences. We have briefly introduced this problem in the perspective of the manuscript*.

### Rewiever report 6 by Yan Boucher (University of Alberta, Canada)

The manuscript presents an ambitious attempt at using novel approaches to investigate large genomic datasets. The methods presented by the authors are able to produce results in agreement with previous findings on the evolution of *E. coli *genomes: that they are involved in frequent LGT and recombination. They also address more specific questions, such as rates of gene transfer for core and shell genes, mobile elements and genes from pathogens versus non-pathogens. What is unique about the approaches used is that they do not assume a single phylogeny, but can tell a story including multiple phylogenies. It is also easy to isolate specific types of genes or organisms from a more complex dataset, allowing the user to answer specific questions. What is difficult about the approaches used here is that they use novel concepts that can be difficult to understand (those linked to clanistics especially) and make the conclusions hard evaluate for most biologists.

*Authors' response: We thank the referee for his comments*.

Specific issues to address:

Abstract:

Problems with the grammatical structure in the results section. This needs to be reviewed by a native English speaker. Language is a bit cavalier, using colloquial terms such as "smoking guns", which are not appropriate for an international audience and only understandable by those with a certain cultural background.

*Authors' response: A native english speaker kindly reviewed the manuscript (Thanks very much Dick!). We replaced "smoking guns" with "strong evidence"*.

Casual language: "(but the RNA viruses, maybe)", "In this paper, we use", "whose main interest is not so much in defining the relative branching order of species". This should be avoided.

*We removed these sentences/words*.

Main text: How were genes determined to be "mobile elements" in their comparison to *E. coli *genomes? The criteria need to be explained.

*Authors' response: We downloaded the genes from plasmids and viruses from the NCBI. Genes from these mobile elements were considered to belong to mobilized or mobilizable gene families*.

The authors should include a legend describing specific network terms such as "betweenness" and "articulation points" or "mélange" or "natives"

*Authors' response: We have described these terms in the main text, where required*.

The authors need to define terms such as "wild genome forest". I would limit the use of new terms to when they are absolutely required

*Authors' response: Wild genome forest is only the name of one of the two forests we studied, reconstructed using all the genes from E. coli *UTI89 (NC007946) *as indicated in M&M. It is not a technical term. We have clarified this issue in the main text*.

A better description of clanistics has to be provided, as it is a new practice. Perhaps some of the materials and method can be included in the main text.

*Authors' response: We have introduced clanistics with some more details in the main text. Readers should also refer to the publications, quoted in the MS*.

The authors should use subtitles to clarify results and highlight interesting findings, such as " similar recombination levels between core and shell genes'

Authors' response: We have added or edited subtitles accordingly. New sections are now called: Using genome networks to detect recent LGT in the E. coli pangenome; Massive tinkering in the evolution of restriction-modification endonucleases; High rates of LGT in E. coli; Pathogenic lifestyle affects the evolution of 30% of the E. coli pangenome; Detection of candidate mobile modules of pathogenicity; Polychromatic quartets reveal high recombination/LGT rates in core and shell genes within E. coli; Preferential exchanges of DNA material between pathogenic E. coli

Table 2 contains too much information and should be presented as graphs or included as supplementary materials

*Authors' response: We have included *Table 2 *as supplementary materials*.

## Supplementary Material

Additional file 1 **Clanistic analyses of *E. coli *pangenome**. Statistics on lateral gene transfer (A) and phenotypic properties (B) inferred from gene trees. Click here for file

Additional file 2 **Organismal information**. Taxonomy and lifestyles of the 30 *E. coli *strains under study. Click here for file
