# Supplementary material for: Of woods and webs: possible alternatives to the tree of life for studying genomic fluidity in E. coli
Source: Biol Direct. 2011 Jul 20;6:39. doi: 10.1186/1745-6150-6-39 (PMC3160433; doi:10.1186/1745-6150-6-39)
Supplement: Additional file 1 — Clanistic analyses of E. coli pangenome. Statistics on lateral gene transfer (A) and phenotypic properties (B) inferred from gene trees. [file 1745-6150-6-39-S1.PDF]

Additional file 1: Summary of the clanistic analysis

(A). Clanistic analysis from a taxonomical perspective of two E. coli centered forests: the wildforest (4065 trees) (with Shigella treated separately from E. coli or as a bona fide E. coli) and the pangenome forest (6129 trees). The EC category is indicated first in the first column, separated from the non-EC category by a '/'. (B). Clanistic analysis of the pangenome forest (6129 trees, with and without accounting for bootstrap support) from a phenotypic perspective. Non trivial perfect clans and slices have > one native for PATH, or each disease (URI, H

A.	*Average Eclan*	*#trees with clans*	*#natives*	*#intruders*	*Average Eslice*	*#trees with slices*	*#intruders*	*Average EMelange*	*#trees with melange*	*#intruders*	*Average pscore*	*#trees without intruder*
**EC/non EC**												
wildforest	0.2468	308	27.42	52.62	0.6585	343	11.74	0.7362	3274	7.894	4.682	140
wild_shig	0.1217	2063	32.26	47.78	0.3538	1089	14.02	0.5507	606	11.66	1.663	307
pangenome	0.2680	731	21.37	1.050	0.639	186	5.942	0.7207	176	6.611	0.3255	5036
**EC/MGE**												
Wildforest	0.2371	302	27.42	0.2502	0.5771	52	2.169	0.3633	66	1.694	0.1565	3645
Pangenome	0.3492	474	21.37	0.8625	0.6379	184	5.919	0.7213	174	6.652	0.2819	5036
**EC/BAC**												
Wildforest	0.24597	313	27.42	51.52	0.6565	358	11.39	0.7321	3244	7.688	4.569	150
wild_shig	0.1125	2142	32.26	46.68	0.3354	1081	14.17	0.5494	497	11.89	1.532	345
Pangenome	1.006e-02	356	21.37	0.1831	0.3349	4	3.6	0.8750	1	5.0	0.05988	5036
**EC/ARC**												
wildforest	1.295e-02	23	27.42	0.01451	0	1	1	NaN	0	NaN	0.00615	4041
pangenome	0	4	21.37	0.002447	NaN	0	NaN	NaN	0	NaN	0.0006526	5036

B.	*Average Eclan*	*#trees with clans (non trivial)*	*#natives*	*#intruders*	*Average Eslice*	*#trees with slices (non trivial)*	*#intruders*	*Average EMelange*	*#trees with melange*	*#intruders*	*Average pscore*	*#trees without intruder*
**PATH/NON PATH**												
Pangenome	0.7703	735 (650)	15.2	7.22	0.8892	547 (547)	6.772	0.9451	4291	6.158	5.163	546
Pang_BV	0.8458	676 (591)	15.2	7.22	0.9192	508 (508)	7.425	0.9519	4389	7.002	6.066	546
**URI/Non URI**												
Pangenome	0.4342	1005 (205)	2.195	20.22	0.8063	1103 (1103)	13.36	0.9121	2974	10.17	2.039	0
Pang_BV	0.6263	867 (67)	2.195	20.22	0.9272	680 (680)	17.73	0.9663	3535	15.84	2.166	0
**HEM/NON HEM**												
Pangenome	0.38984	872 (187)	3.173	19.24	0.5476	1458 (1458)	11.90	0.7792	2854	10.09	2.357	1
Pang_BV	0.6990	762 (77)	3.173	19.24	0.9340	367 (367)	18.09	0.972	4055	16.12	2.994	1
**GAS/NON GAS**												
Pangenome	1.912e-02	4254 (6)	0.775	21.64	0.6258	98 (98)	32.80	0.7261	64	25.55	0.7703	0
Pang_BV	1.912e-02	867 (67)	0.775	21.64	0.8736	680 (680)	41.32	0.9235	3535	37.66	0.7719	0
